# The microstructure and mechanical properties of microwave-heated lunar simulants at different input powers under vacuum

**DOI:** 10.1038/s41598-023-29030-z

**Published:** 2023-01-31

**Authors:** Sungwoo Lim, Giulia Degli-Alessandrini, James Bowen, Mahesh Anand, Aidan Cowley

**Affiliations:** 1 School of Physical Sciences, The Open University, Milton Keynes, MK7 6AA UK; 2 School of Engineering and Innovation, The Open University, Milton Keynes, MK7 6AA UK; 3 European Astronaut Centre, Köln, Germany

**Keywords:** Civil engineering, Mechanical properties, Composites

## Abstract

To achieve a sustainable human presence on the Moon, it is critical to develop technologies utilising the local resources (a.k.a. in-situ resource utilisation or ISRU) for construction and resource extraction. In this study, we investigate the viability of microwave heating of two lunar soil simulants (JSC-1A and OPRH3N) under vacuum conditions, to simulate a lunar surface environment compared to previous studies performed at atmospheric pressure. All simulants are thermally treated in a bespoke 2.45 GHz microwave apparatus using three input powers: 1000 W, 600 W and 250 W. The microstructures and mechanical properties of the microwaved samples are analysed to identify their potential applications. Our key findings are: (i) higher input powers generate materials in shorter fabrication times with higher mechanical strengths and higher yields despite the same total energy input; (ii) the microstructures of the microwaved samples under vacuum are very different from those under atmospheric conditions due to the widespread vesicles/bubbles; and (iii) different heating rates caused by different input powers can be utilised for specific ISRU purposes: higher input powers for extra-terrestrial construction and lower input powers for resource extraction. Findings from this study have significant implications for developing a microwave-heating payload for lunar ISRU demonstration missions.

## Introduction

As part of Europe’s space exploration strategy, the European Space Agency (ESA) Council created the European Exploration Envelope Programme (E3P) in 2016, now called *Terrae Novae*, meaning ‘New World’. Terrae Novae 2030+ is an exploration strategy encompassing three ESA exploration destinations: Low Earth Orbit (LEO), the Moon and Mars. In the Moon and Mars environment, habitat construction and resource extraction are considered two of the five key elements of the strategic roadmap for human presence on the Moon and Mars. A key technology most likely to be employed in such lunar construction processes is a robotic 3D Printing platform^1^, utilising sintered/molten lunar soil as the construction material because of its uncomplicated and autonomous operation. Due to the efficiency of volumetric heating intrinsic to the microwave process, microwave sintering/melting is considered a viable fabrication method for a 3D Printing platform. This technique requires ~ 23% of the energy compared to laser sintering and reduces fabrication times, as discussed in^1–3^.

This paper reports on the outcome of a series of microwave heating experiments on a lunar mare soil simulant JSC-1A and a lunar highland soil simulant OPRH3N. Both simulants were subjected to microwave heating under vacuum conditions (10^–4^ Pa) to mimic the lunar environment. This research builds upon the previous experiments conducted under atmospheric conditions^3^. Samples were melted using 1000 W, 600 W and 250 W input powers, using the same bespoke 2.45 GHz microwave apparatus described in^3^. The objective is to investigate how the microstructures and mechanical strengths of the sintered/molten samples are affected by vacuum, keeping all other experimental parameters the same as in the previous experiments.

The findings from this work will be used towards developing a microwave-heating payload, currently supported by the UK Space Agency (UKSA) and European Space Agency (ESA), that could potentially be part of an *In-Situ* Resource Utilisation (ISRU) Demonstrator mission for heating-based resource extraction and habitat construction using 3D printing technology. More specifically, the findings of this work will contribute toward determining the optimal input power for microwave sintering/melting of lunar soil for 3D printing; and the minimum input power for extraction of lunar resources such as oxygen, water and iron from the soil.

## Results

**Figure 1:**
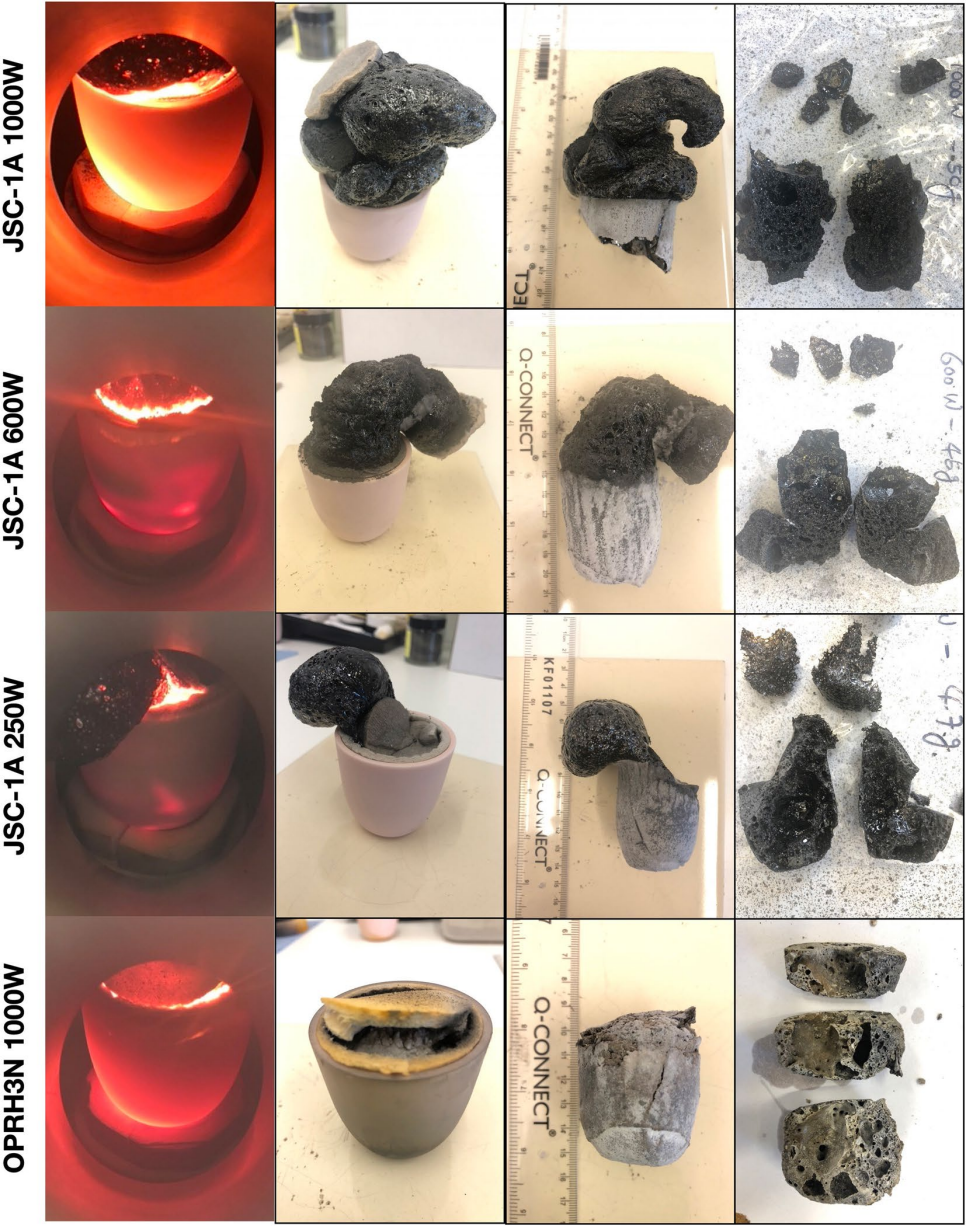
Selection of microwaved samples of lunar simulants JSC-1A and OPRH3N, heated with a bespoke microwave apparatus using different microwave input powers (1000 W, 600 W and 250 W). Images from left to right show the heated, cooled, and sliced products of microwave heating.

**Figure 2:**
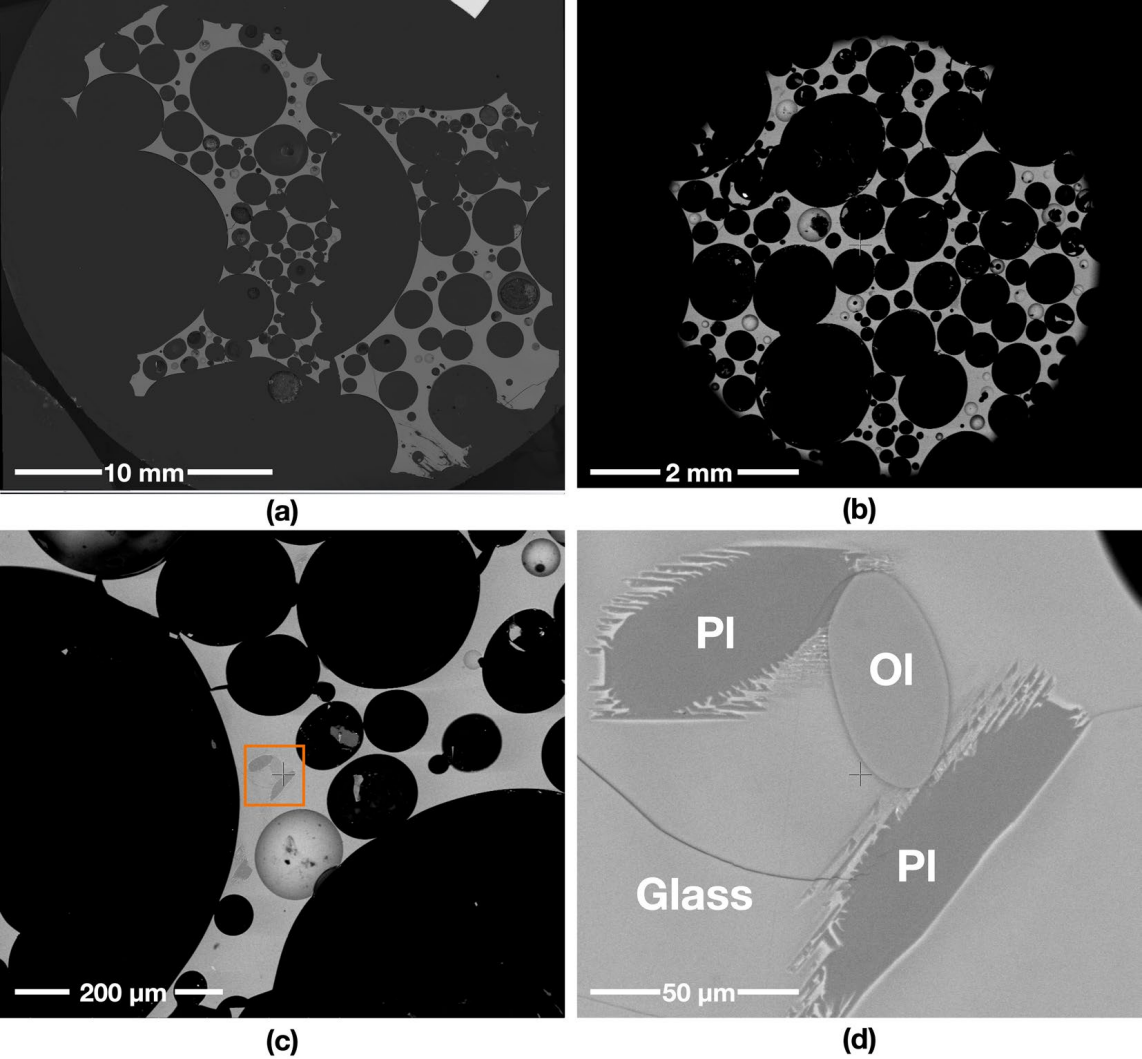
Backscattered Electron (BSE) images of the JSC-1A sample microwave heated in vacuum, using 1000 W input power. (**a**) and (**b**) show the sample with abundant bubbles (black, round features). The grey areas are glass, while the black areas are bubbles. The sample is almost fully molten, with only a few exceptions. (**d**) is a detail of (**c**), showing a few mineral grains: olivine (Ol) and plagioclase feldspar (Pl), in the glass of the fully molten area. The plagioclase grains crystallise with acicular projections, reacting with the molten glass.

**Figure 3:**
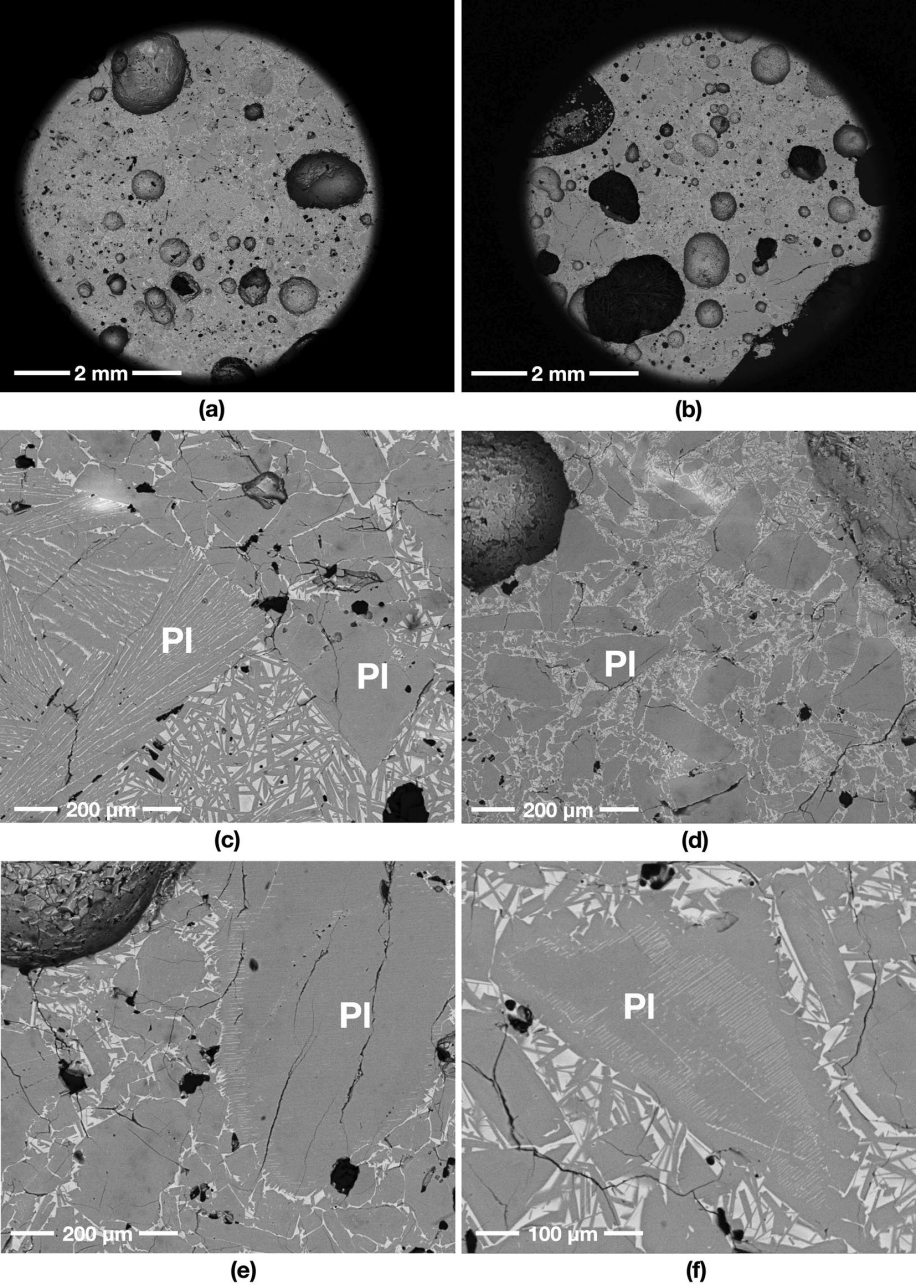
BSE images of the OPRH3N sample microwave heated in vacuum using 1000 W input power. (**a**) and (**b**) show an overview of the sample, while (**c**) and (**d**) indicate different crystallisations of the heated plagioclase feldspar (Pl) grains. (**e**) and (**f**) show the feldspathic glass creation process due to melting and recrystallising a mix of feldspar and pyroxenes.

**Figure 4:**
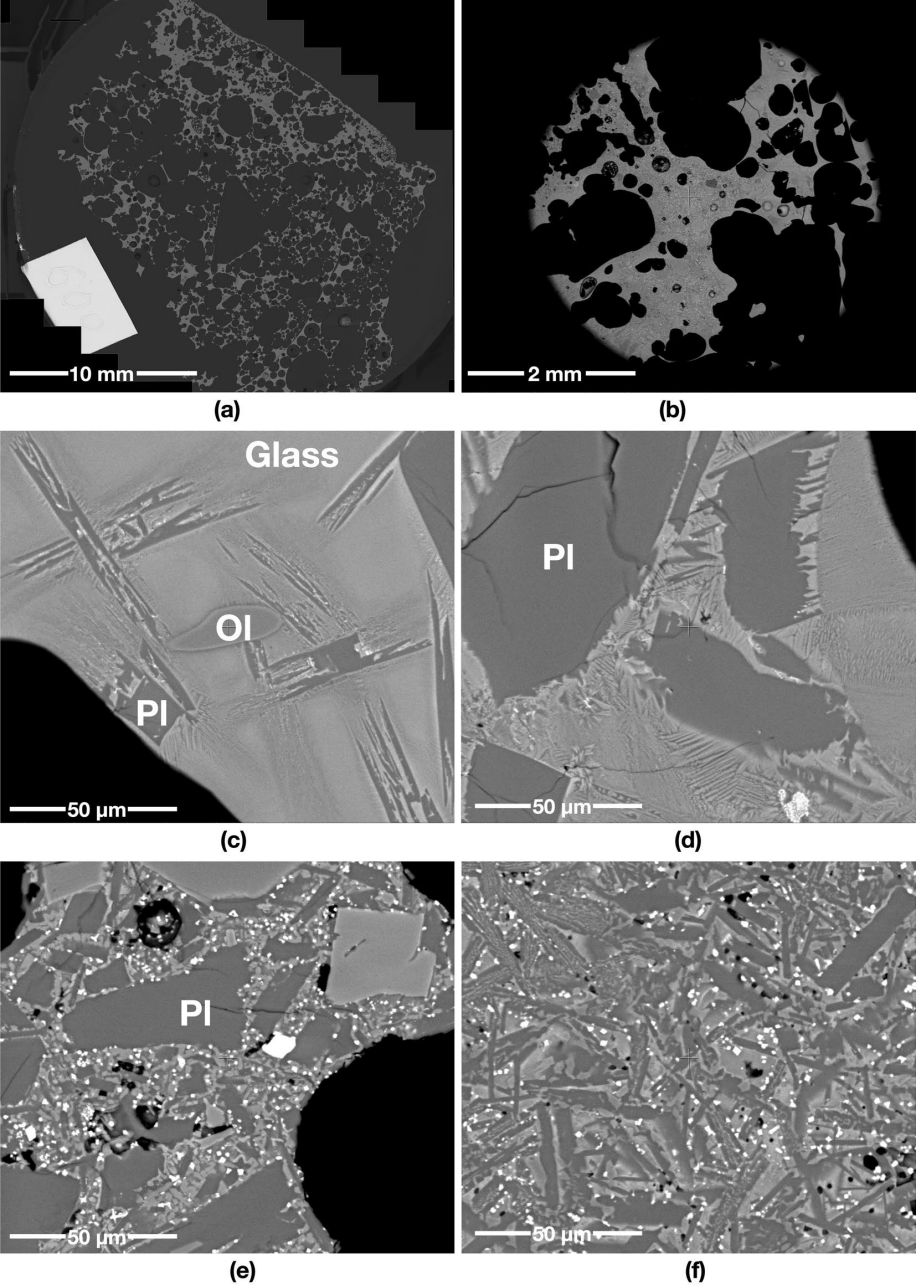
BSE images of the JSC-1A sample microwave heated in vacuum using 600 W input power. (**a**) and (**b**) show an overview of the sample displaying abundant voids from the original powder porosity combined with bubbles created by released volatiles. (**c**) and (**d**) show the microstructure of the partially molten areas, where plagioclase (Pl) reacts with molten glass and crystallises with acicular projections in the partially molten area. (**e**) and (**f**) show the sintered areas, with pervasive nucleation of SP_Fe_ particles/needles in the glassy matrix.

**Figure 5:**
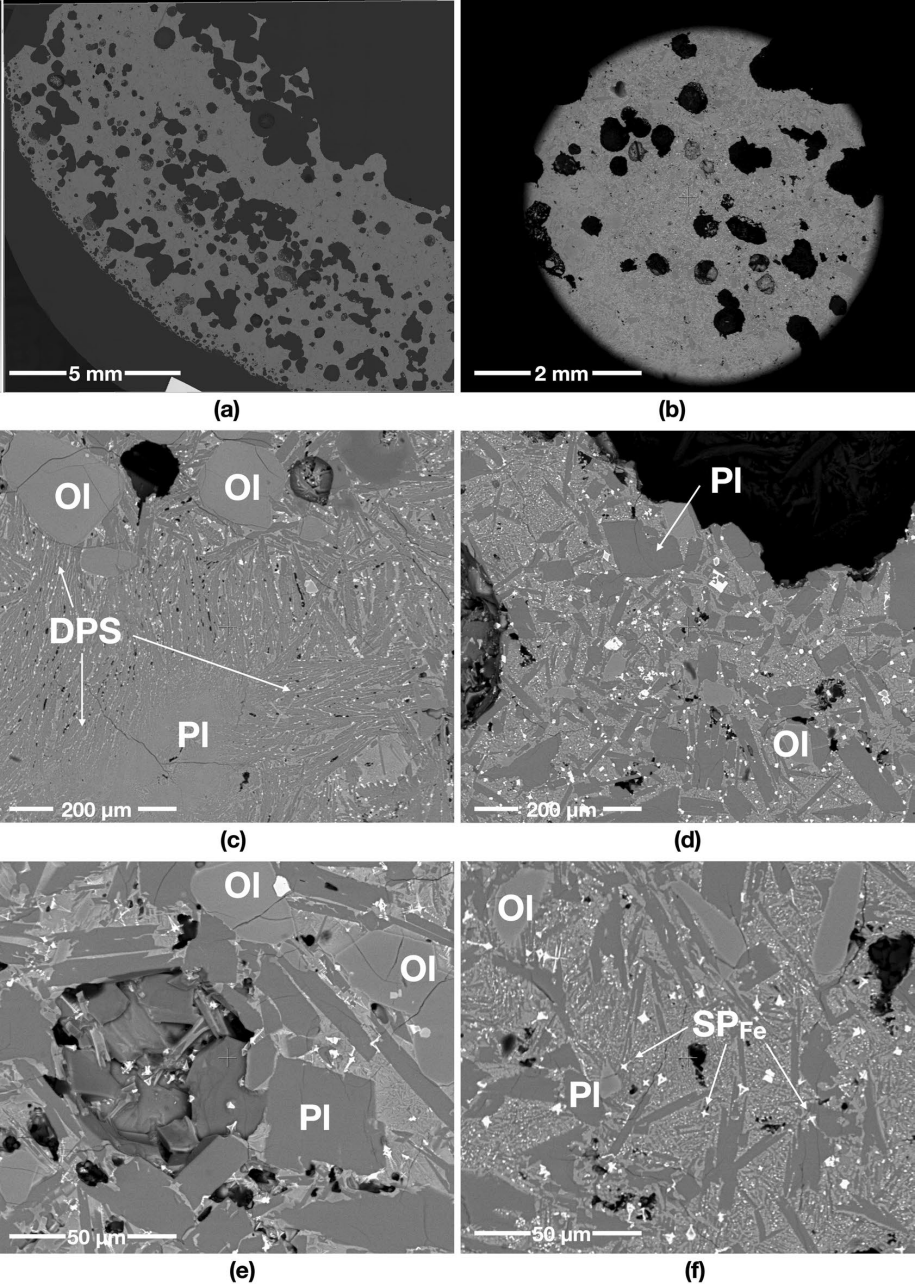
BSE images of the JSC-1A sample microwave heated in vacuum using 250 W input power. (**a**) and (**b**) show an overview of mostly sintered areas with abundant but fewer voids than the 600 W input power samples (see more details in Fig. 7). The partially molten and sintered areas in (**c**) to (**f**) have abundant SP_Fe_ particles with some dendritic patterned silicates (DPS) over the entire area. (**c**) indicates that most plagioclase (Pl) grains in the partially molten area are dissolving and crystalising, while (**d**) and (**e**) show that most plagioclase (Pl) and olivine (Ol) grains in the sintered area still preserve their original sub-angular shapes. (**f**) depicts the skeletal growth morphologies of SP_Fe_ classified as a cruciform type.

A starting mass of 50 g of powder simulants JSC-1A and OPRH3N were microwave heated using three different input powers. For JSC-1A, three samples were heated with 1000 W for 900 s, 600 W for 1500 s and 250 W for 3600 s. However, OPRH3N was heated with 1000 W for 3600 s only because the current microwave setting is more optimal for JSC-1A, resulting in a lower heating performance of OPRH3N at lower input power (Fig. 1). After the experiment, the samples yielded a sintered/molten and solidified mass of 100% (1000 W, JSC-1A), 90% (600 W, JSC-1A), 94% (250 W, JSC-1A) and 98% (1000 W, OPRH3N). Note that *yield* here is defined as the mass of the sintered/molten part out of the original 50 g of mass of the untreated powdered raw material. As observed in the previous experiments^3^, all microwaved samples display three distinct microstructural areas (fully molten, partially molten and sintered), depending upon the extent of heating experienced in each case. Under vacuum conditions, only the JSC-1A samples with 1000 W input power shows fully molten (mostly glass) areas (Fig. 2). The other samples mainly show sintered and partially molten areas (Figs. 3, 4 and 5). The partially molten areas show a predominance of glass with some relic mineral grains and the newly formed minerals from the melt with abundant bubbles. The sintered areas in Figs. 3, 4 and 5 comprise a mixture of (i) un-melted mineral grains such as olivine and plagioclase feldspar showing evidence of chemical alterations at the grain-glass interface, (ii) a glassy matrix containing newly formed minerals and iron-rich spinel (SP_Fe_) particles, and (iii) a few vesicles/bubbles, which are similar with the samples in the previous experiments.

### 1000 W input power

The JSC-1A sample melted with a 100% yield rate (50 g out of 50 g) within the given time (900 s, i.e., 15 min). The sample structure is mostly fully molten glass with abundant bubbles due to volatile release during heating (Fig. 2), resulting in a highly porous structure (Fig. 1). The rate of the supplied energy at 1000 W is much higher than that of other input powers despite the same total energy (900 kJ) being provided for all. This causes a much higher heating temperature, producing a fully molten and highly porous structure with abundant bubbles.

Although the highland simulant was not analysed in the previous experiment, we have added the OPRH3N sample in this study because the most imminent lunar mission location is a highland area, i.e., Shackleton Crater in the South lunar pole area. Due to the different chemical composition, Fe (2.69 wt% of FeO and 0.0 wt% of Fe_2_O_3_) is much lower than JSC-1A (7–7.5 wt% of FeO and 3–4 wt% of Fe_2_O_3_), the OPRH3N sample has much lower microwave heating performance. Thus, we had to increase the total energy input—four times longer than the JSC-1A sample, i.e., 3600 kJ. To compare the microstructure of microwaved OPRH3N sample, we heated the sample with 1000 W input power only because the sample was not sintered/melted with lower input powers, 600 W and 250 W.

**Figure 6:**
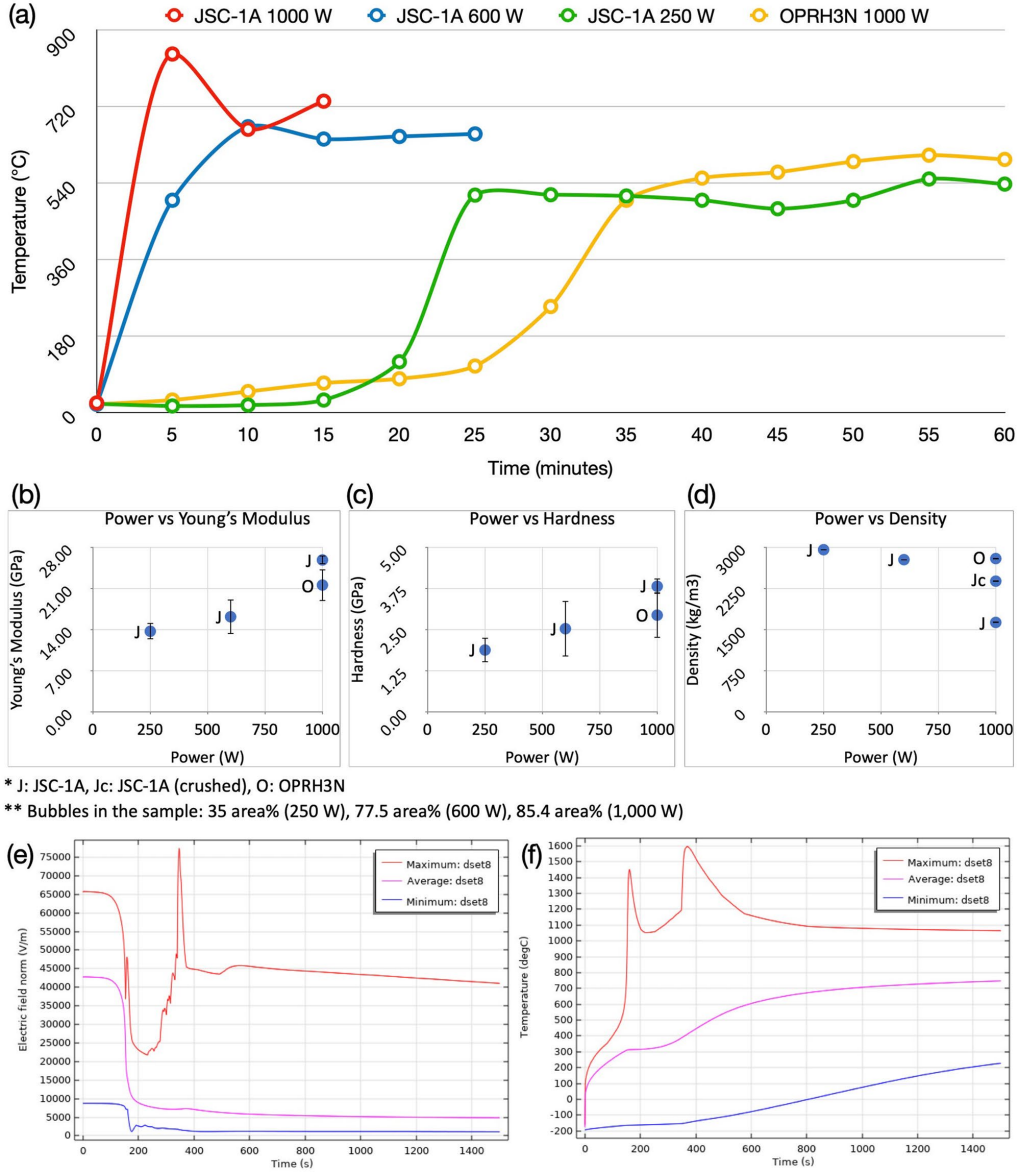
Heat performance and mechanical properties of the microwaved samples under vacuum conditions. (**a**) Time–temperature curves of microwaved heated samples, using different input powers, for a total energy dose of 900 kJ for JSC-1A and 3600 kJ for OPRH3N. Note that the temperatures correspond to the outer surface temperature of the crucible. (**b**) Young’s Modulus, (**c**) Hardness and (**d**) True Density of samples microwaved with different input powers. J: JSC-1A; Jc: crushed JSC-1A; O: OPRH3N. Note that (**e**) and (**f**) show the simulation result of lunar mare soil heating with the starting temperature of − 193.15 °C (80 K). (**e**) Three Electric Field (EF) curves in the sample. The red and blue curves show the maximum and minimum EF points in the sample, while the purple curve shows the average EF of the sample. As shown in (**f**), the strength of the EF in the sample directly affects the microwave heating performance of the sample. (**f**) Three temperature curves of the sample. The red and blue curves show the maximum and minimum temperature points in the sample, while the purple curve shows the average temperature of the sample.

The OPRH3N sample melted with a 98% yield rate (49 g out of 50 g) within 3600 s, i.e., 60 min. The sample was partially molten with much fewer vesicles/bubbles than the JSC-1A sample. It also shows unusual microstructures of plagioclase feldspar grains (Fig. 3e and f). A few plagioclase feldspar grains present an exciting phenomenon contrary to the iron-rich spinel (SP_Fe_) particles’ exsolution from olivine grains observed in the previous experiments (Figs. 4, 5, 6 in^3^). The brighter hatching lines in the plagioclase grains contain Mg (1–4 wt%) and Fe (2–9 wt%), which must have resulted from the melting of mafic phases and remobilisation of Fe (and to some extent Mg) from the melted glass throughout the sample along grain boundaries, exploiting all weakness/structures to invade/diffuse. This feature depicts the feldspathic glass crystallisation process, resulting from the melting and recrystallisation of a mix of feldspar and pyroxenes. In Fig. 3e and f, the core of the plagioclase feldspar (inside the brighter hatching lines) is the relic plagioclase feldspar grain, and the brighter hatching lines on the boundary of the relic depict the trace of the melted glass intrusion into the grain. The outer part of the hatching lines is the melted and recrystallised plagioclase feldspar. Because Al and Ca are more sluggish to diffuse out of the feldspar structure, this feature indicates that the sample has experienced a slow and longer heating process than the JSC-1A sample.

The highland simulant OPRH3N did not sinter/melt at 600 W and 250 W input powers within the heating time. This indicates that lunar highland soils require higher input powers to trigger thermal runaway than lunar mare soils.

### 600 W input power

The JSC-1A sample melted with 90% of the yield rate (45 g out of 50 g) at the given heating time (1500 s, i.e., 25 min) with a total energy of 900 kJ. Most of the 600 W sample is sintered (Fig. 4e and f), and a few partially molten areas containing abundant relict mineral grains can still be seen (Fig. 4c and d). The sintered areas have many irregular-shaped voids (Fig. 4a), possibly from the original powder porosity combined with newly created bubbles. The original fragments of JSC-1A minerals are still visible in these areas (Fig. 4e). The sample is also characterised by the pervasive nucleation of iron-rich spinel (SP_Fe_) particles in the glassy matrix (Fig. 4e and f), which were broadly observed in the 400 W and 250 W samples microwaved under atmospheric conditions (Figs. 5 and 6 in^3^). Plagioclase grains are still subhedral in shape with no growth of needles (Fig. 4e and f). At the same time, they show the growth of needles in the partially molten areas indicating new crystallisation of plagioclase grains from the melt (Fig. 4c and d). The olivine grain still preserves its shapes; however, the change of the boundary colour with the wobbly boundary shape indicates an initial reaction with the surrounding molten glass. A similar feature was observed in the 1000 W and 800 W samples microwaved under atmospheric conditions (Figs. 2 and 3 in^3^). This indicates that the 600 W sample under vacuum conditions experienced higher temperatures than the 1000 W and 800 W samples under atmospheric conditions.

### 250 W input power

The 250 W sample was mostly sintered with a 94% yield (47 g out of 50 g) at the given heating time (3600 s, i.e., 60 min). The sintered areas contain many SP_Fe_ particles (Fig. 5d and f), similar to the 250 W sample microwaved under atmospheric conditions (Figs. 5 and 6 in^3^). As mentioned in^3^, this feature can be observed in Si-rich andesite, where abundant spinel particles nucleate in the glassy matrix under prolonged heating conditions (10 °C/min to 1300 °C)^4^. Some SP_Fe_ particles show complex geometric patterns (Fig. 5f), identified as the skeletal growth morphologies of titanomagnetite classified as a cruciform type^5^. SP_Fe_ particles in 250 W under vacuum conditions are larger crystals than those observed in the 600 W sample under vacuum conditions. However, none of the olivine grains shows any trace of SP_Fe_ particle aligned with the darker rims of the olivine grains, as observed in the partially molten areas in the 250 W samples microwaved under atmospheric conditions (Figs. 5 and 6 in^3^). This indicates that the 250 W samples under vacuum conditions experienced lower temperature than the 250 W samples under atmospheric conditions.

From the comparison of samples heated under atmospheric and vacuum conditions, it is clear that both simulants display very different structures at the same input powers due to the widespread bubbles in the samples heated under vacuum conditions.

### Sample temperature during microwave heating

Figure 6a shows the temperature curves of the four samples heated under different input powers with the corresponding duration of heating for a total energy dose of 900 kJ for JSC-1A and 3600 kJ for OPRH3N samples. Note that the recorded temperatures are for the outer crucible surface instead of the sample surface in this experimental setup. Furthermore, the hotspot occurred in the samples’ core, and the samples were surrounded by ceramic paper preventing thermal shock to the alumina crucible, resulting in significant thermal insulation. Thus, the recorded temperatures shown in Fig. 6a are much lower than what the samples experienced, as the hotspot temperature caused by thermal runaway is around 1700–2200 °C^6^.

Similar to the microwaved samples under atmospheric conditions in^3^, Fig. 6a shows that, for simulant JSC-1A, higher input powers reached higher peak temperatures in a shorter time. In contrast, the peak temperatures of lower input powers were reached more slowly. All four samples experienced thermal runaway with bright hotspots on the crucible surface. Regardless of the absolute temperatures experienced by the samples, the temperature curves of the four samples display similar heating trends, including thermal runaway resulting in a radical temperature increase. These results are similar to those of the samples microwaved under atmospheric conditions. Figure 6a shows a flattening of the temperature curves towards the end of their heating cycles, which indicates a lower efficiency of microwave heating, possibly caused by the decrease of the electric field in the sample. This cause has been verified through a Multiphysics simulation of the microwave heating behaviour of lunar mare soil shown in Fig. 6e and f. The maximum electric field curve indicates that the electric field in the sample fluctuates with temperature increase, i.e., it dramatically decreases when the sample is heated (after 150 s), spikes with thermal runaway (after 350 s), and decreases again up to a certain level (around 800 s) shown from the maximum curve in Fig. 6e. This decrease in the electric field causes poor heating performance in the later stage of microwave heating. The difference between the three curves in Fig. 6f indicates that the hotspot formed as a result of thermal runaway is relatively small as the high electric field could not be sustained over a prolonged period, which means that the hotspot-induced heat couldn’t penetrate the entire sample, resulting in a significant temperature difference in the simulated sample. This is why the delta (Δ) between the maximum and minimum EF and temperature curves are significant, e.g., the hotspot temperature reaches 1600 °C while some part of the sample surface temperature is lower than − 100 °C, as shown in Fig. 6f. This is because (i) the hotspot size is relatively small, (ii) the high electric field cannot be sustained for a more extended period, and (iii) the thermal conductivity of lunar regolith is very low, decreasing the heat transfer from the hotspot in the core to the surface of the sample. The decrease of the electric field in the sample is related to the change of material properties by temperature increase; however, the exact cause needs to be investigated further.

Note that the current microwave chamber design and frequency allow better heating performance for lunar mare soil simulant JSC-1A than OPRH3N as the hotspot occurs in the sample core properly, even though the noticeable plasma effect reduces the heating performance. Adjusting the resonant frequency of the microwave chamber could optimise the heating performance of specific materials; thus, the current microwave heating performance of lunar highland soil could be further improved through future work.

### Physical and mechanical properties

The experimental results reveal that even the 250 W JSC-1A sample experienced thermal runaway and sintered/melts under vacuum conditions. This differs from the JSC-1A microwaved JSC-1A samples under atmospheric conditions using 250 W input power, where most of the starting material did not allow sinter/melt, as proven by the low yield (44%, 22 g out of 50 g)^3^. The sample’s physical and mechanical properties under atmospheric and vacuum conditions are summarised in Table 1 for direct comparison.

**Table 1:** Comparison of the samples’ physical and mechanical properties under atmospheric and vacuum conditions (JSC-1A).

Input power (W)	Physical and mechanical properties (atmospheric^3 ^/ vacuum)			
	Young’s Modulus (GPa)	Hardness (GPa)	Density (kg/m^3^)	Porosity (volume%)
1000	70.00 / 25.85	7.73 / 3.82	3020 / 2383*	N.A. / 85.4
800	76.30 / N.A	7.75 / N.A	2980 / N.A	N.A
600	64.80 / 16.18	6.78 / 2.53	2810 / 2775	N.A. / 77.5
400	49.30 / N.A	5.52 / N.A	2810 / N.A	N.A
250	46.20 / 13.70	5.04 / 1.88	2830 / 2957	N.A. / 35.0

*The density of the crushed sample. The density of the original sample is 1630 kg/m^3^.

#### Young’s modulus and hardness

The mean Young’s Modulus (25.85, 16.18, 13.70 GPa) and the hardness (3.82, 2.53, 1.88 GPa) of the JSC-1A samples (1000 W, 600 W and 250 W) are shown in Fig. 6b and c are less than half of the Young’s Modulus (70.0, 64.8, 46.2 GPa) and the hardness (7.73, 6.78, 5.04 GPa) of the JSC-1A samples melted under atmospheric conditions^3^. The difference of the microwave chamber pressure, i.e., atmospheric vs vacuum (10^–4^ Pa) conditions, led to a significant impact on the mechanical properties of the microwaved samples. Despite the much lower mechanical properties of vacuum samples, likely caused by the high porosity due to abundant vesicles/bubbles, the samples still show positive correlations of the input power vs Young’s Modulus, and input power vs Hardness (Fig. 6b and c). Higher input power gives a higher heating rate, effectively resulting in a homogeneous melting with fewer impurities and less sintering, thus achieving higher hardness. Interestingly, the mean Young’s Modulus (21.58 GPa) and the Hardness (2.94 GPa) of the OPRH3N 1000 W sample are lower than the JSC-1A 1000 W sample, probably caused by the poorer heating performance of OPRH3N (Fig. 6b and c).

#### True density

The true density analysis of vacuum samples indicates that the 250 W input power produces the highest mean true density, i.e., 2957 kg/m^3^, compared to 2775 kg/m^3^ for 600 W, and 1630 kg/m^3^ (2383 kg/m^3^ when crushed) for 1000 W (Fig. 6d). For comparison, the true density of atmospheric samples is 2830 kg/m^3^ for 250 W, 2810 kg/m^3^ for 600 W and 3020 kg/m^3^ for 1000 W^3^. Nevertheless, the 600 W and 250 W samples still have much higher true densities than the heavyweight concrete (> 2600 kg/m^3^). However, as mentioned in^3^, the density may not directly correlate with hardness because the hardness measurement only applies to the material surface. For example, the mean bulk density of lunar highlands and mare rock is around 2510 ± 20–2840 ± 40 kg/m^3^ with a porosity range from 2.2 to 11.5% and 3010 ± 40–3270 ± 50 kg/m^3^ with a porosity range from 1.8 to 10.3%, respectively^7^. It is shown that even with the high porosity and mostly sintered and partially molten microstructures, the highland sample (OPRH3N)’s density is similar to the mean bulk density of lunar highland rock as it developed much fewer bubbles compared to the equivalent JSC-1A.

**Figure 7:**
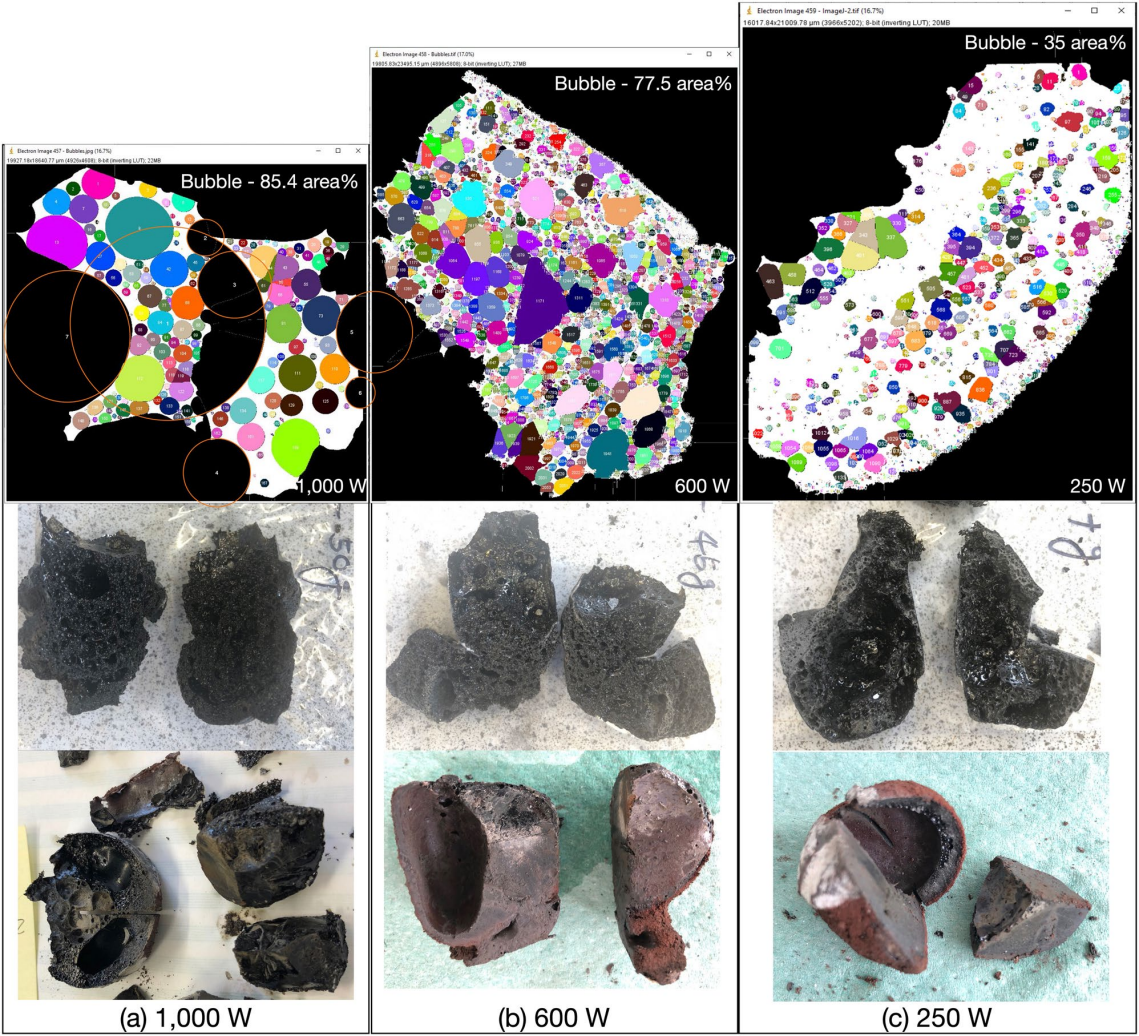
Bubble quantity measurement of the JSC-1A samples—(**a**) 1000 W sample with 85.4% of bubble over the glass, (**b**) 600 W sample with 77.5% of bubble over the glass, (**c**) 250 W sample with 35% of bubble over the glass. The bubble quantification images are on the same scale. The 1000 W sample doesn’t correctly represent the abundant giant bubbles with the current sample as it does not include the full size of some giant bubbles on the sample edges. Thus, such bubbles (orange circled) in the 1000 W sample were also added for the calculation. The sliced sample images show the inner structure of each sample. The microwaved samples under atmospheric conditions have a giant bubble in the core where a hotspot occurred (Bottom images), while the samples under vacuum have abundant tiny and micro-size bubbles widespread (Middle images).

The foam-like structures of 1000 W and 600 W JSC-1A samples result from widespread bubble formation during microwave melting/sintering under vacuum conditions (Fig. 7). The image analysis of the sample cross-section indicates that bubbles constitute 85.4 area% of the 1000 W sample, 77.5 area% of the 600 W sample and 35 area% of the 250 W sample. The abundance of bubbles in vacuum samples seems to negatively correlate with the sample’s true density, unlike the samples heated under atmospheric conditions, i.e., the higher the bubble to glass percentage, the lower the true density of the sample (Fig. 6d). The 250 W JSC-1A sample shows much fewer vesicles/bubbles (35 area% of bubbles over glass); consequently, the 250 W JSC-1A sample has a higher true density. The possible reasons for this phenomenon are as below.

- The 1000 W and 600 W of the JSC-1A samples had experienced thermal runaway (higher temperature) and melting in a relatively short time. This may have released the volatiles suddenly, resulting in large-sealed bubbles. The melt was also quenched in a short amount of time under vacuum conditions, which may have caused the volatiles to be trapped in the glass in the form of bubbles. Because of the bubbles showed in Fig. 7, 1000 W of the JSC-1A sample shows a much lower true density than 600 W and 250 W products. To minimise the effect of porosity, the 1000 W JSC-1A sample was crushed and re-measured for its true density. This led to an increase in its true density but still lower than 600 W and 250 W samples, which means there are still many micro-pores remaining in the crushed sample.
- On the other hand, the released volatiles from the 250 W JSC-1A and 1000 W OPRH3N samples could have had enough time to escape when the sample is sintered/melted; thus, presenting fewer bubbles in the samples.

## Discussion

The primary purpose of this work is to understand the microwave heating behaviour of lunar soil under vacuum conditions by measuring the microstructure and mechanical properties of the samples after heat treatment using 1000 W, 600 W and 250 W input power.

### Microstructural analysis

The Scanning Electron Microscope (SEM) analysis of each sample indicates that microwave input power plays a vital role in each sample's overall microstructure and mineral makeup. The fabricated samples produce distinct microstructural areas, classified in^3^ as sintered, partially-molten and fully molten areas. The most apparent difference between vacuum and atmospheric samples is the dominance of bubbles in the vacuum samples, which suggests a different release of volatiles and other behaviour of the gaseous phase when melting in a vacuum.

The JSC-1A sample under 1000 W of input power produces a homogeneous glass microstructure with abundant bubbles throughout the sample volume, possibly caused by the following reason. Under atmospheric conditions, the chamber pressure was increased with the sample heating; thus, the released volatiles was trapped in the core of the sample as a large bubble where the hotspot occurred. On the other hand, under vacuum conditions, the continuous low chamber pressure generated by a continuously operating vacuum pump caused the volatiles to be released outside the sample. Due to the basaltic molten glass’s viscosity, most volatiles is trapped in small and micro-size bubbles and form a foam-like structure instead of creating a large bubble (see Fig. 7). The 600 W input power sample produced a slightly inhomogeneous microstructure compared to 1000 W, with partially dissolved/reacted minerals. In comparison, 250 W of input power produced pervasive neo-crystallisation of square-shaped SP_Fe_ and DPS. Regarding the bubble size and shape, the 600 W and 250 W samples are distinctive from the 1000 W sample. While the 1000 W sample shows bubbles of circular shapes with sizes ranging from 272.33 mm^2^ to 49 μm^2^ (175 bubbles, average 3.112 mm^2^), the 600 W sample has abundant irregular-shaped vesicles and a few circular-shaped bubbles of sizes ranging from 9.00 mm^2^ to 16 μm^2^ (2058 bubbles, average 0.099 mm^2^). Likewise, the 250 W sample has smaller vesicles/voids/bubbles than the 600 W sample, ranging in size from 1.73 mm^2^ to 16 μm^2^ (1200 bubbles, average 0.056 mm^2^). This also indicates that the samples with higher input power experienced higher temperatures with an abundant and sudden gas release.

Considering 3D printing or other manufacturing/construction applications, these microwaved samples under vacuum prove that higher input powers are still more efficient for fabrication and are microstructurally more homogeneous than lower input powers. However, abundant bubbles due to vacuum conditions in the microwave chamber cause a significant decrease in the bulk density (not true density) and mechanical strengths (Young’s Modulus and Hardness) across all input powers for the lunar mare soil simulant JSC-1A samples, compared to the equivalent JSC-1A samples fabricated at ambient pressures^3^. Interestingly, the lunar highland soil simulant OPRH3N sample with 1000 W requires four times more energy (3600 kJ, 60 min heating) to achieve the onset of sintering/melting. The OPRH3N sample also has lower mechanical strength than the JSC-1A 1000 W, despite higher bulk density (Fig. 6b,d). Because of the significantly lower Fe contents in OPRH3N compared with JSC-1A, which directly affect microwave energy absorption, the heating performance of microwave energy to OPRH3N is lower than that of JSC-1A. Our preliminary computational simulation indicates that the difference in the resonant frequency between lunar highlands and mare soils is over 200 MHz, which affects the heating performance significantly. These results indicate that the resonant frequency of the microwave chamber needs to be adjusted by materials, as the current microwave setting is more optimised for JSC-1A. Thus, more studies on the effect of resonant frequency are planned in future work.

All samples containing the sintered and partially-molten areas look similar, regardless of input power. More specifically, the matrix between grains has melted into a glass which subsequently crystallised into a micro-crystalline matrix of iron-rich spinel (SP_Fe_) particles/needles and plagioclase (Fig. 4 and 5). SP_Fe_ particles/needles are widespread in the matrix and in and around olivine grains, most abundant in sintered areas with low input powers, i.e., 250 W. The element weight percent (el-wt%) of the SP_Fe_ particles/needles shows a higher concentration of Fe and Ti, ranging from 69.3 to 79.58 el-wt% (average 75 el-wt%) of Fe, and from 6.53 to 14.41 el-wt% (average 10.4 el-wt%) of Ti. These iron/titanium-rich spinel particles are mostly some forms of iron oxide, e.g., iron oxide (FeO, 77.73 wt% of Fe), haematite (Fe_2_O_3_, 69.94 wt% of Fe) and magnetite (Fe_3_O_4_, 72.36 wt% of Fe). Irrespective of the exact mechanism(s) of formation, the abundance of SP_Fe_ particles/needles in low input power samples suggests that microwave heating with low input power could be utilised as an effective iron beneficiation method. This means it might be easier to extract iron from partially molten and sintered soil under microwave heating than from untreated soil because the iron-rich spinel (SP_Fe_) particles are effectively separated from minerals containing Fe.

### Young’s modulus and hardness

The heating performance of each experiment under vacuum conditions seems higher than that under atmospheric conditions, particularly with lower input powers. For example, the yield of 250 W samples under vacuum and atmospheric conditions is 94% and 44%, respectively. As shown in Fig. 1, all JSC-1A samples were fully sintered/melted, and there was no untreated residue powder in the crucible. This indicates that the hotspot heat effectively reached the sample surface, resulting in fully sintered/melted. However, the microwaved samples under vacuum showed lower mechanical properties (Young’s Modulus and Hardness), possibly caused by the abundant bubble formation. Gas release during microwave heating resulted in higher porosity, which posed challenges in measuring the mechanical properties. Although nanoindentation was used successfully for the samples heated under atmospheric conditions^3^, the samples heated under vacuum conditions tended to fracture and crumble upon the nanoindenter tip making contact. Successful measurements were only achieved in a small number of regions, despite numerous attempts. As was discussed earlier, the input power has a positive linear relation to the mechanical strengths under both atmospheric and vacuum conditions (Fig. 6b–d). This suggests that samples fabricated with higher input powers are stronger than those fabricated with lower input powers. It follows that higher input powers are preferred for 3D printing construction, and oxygen extraction, and lower input powers are preferred for iron beneficiation.

### True density

Generally, the solubility of gases decreases with temperature increase, and the expansion of gases at higher temperatures means that bigger bubbles may form, even though the quantity of gas itself may be the same. This means more bubbles are visible in the samples heated with higher temperatures, corresponding to the samples fabricated with higher input powers (1000 W). Moreover, the solubility of gases depends on pressure. According to Henry’s law^8^, the solubility of gases in the liquid phase is less when the pressure is lower. Thus, at lower pressure, less gas is stable in the melt. According to the experiment results, we believe that the combined effect of low pressure and high temperature at 1000 W promotes the release of the gas phase from the melt, resulting in an abundance of bubble formation that remains trapped by rapidly cooling melt films. This phenomenon is directly opposite to the results under atmospheric conditions reported in^3^. At lower input powers, results show that the melting is less efficient (i.e., lower volumes of melt produced) and that the peak temperature is lower. Under these conditions, the samples may have released less gas over a longer period. The slower melting rate may have allowed the produced gas to escape in the chamber through the powder's pre-existing porosity. The absence of large bubbles seems to correlate with higher bulk densities, i.e., low input power samples produce higher bulk densities while high input power samples produce lower bulk densities.

## Summary

The work presented in this paper revealed a few notable findings, as summarised below.

- The heat performance of microwave heating under vacuum conditions is much better than under atmospheric conditions because the sample yields are much higher, and there was no untreated residue powder in the crucible.
- Higher input powers (1000 W) generate homogeneous glass samples with abundant bubbles. Although higher input powers allow higher mechanical strengths and higher yields in less fabrication time, the abundant bubbles cause foam-like structures affecting the mechanical properties and density, requiring additional treatments to be directly used as a construction material. For example, the microwaved lunar soil may need to be continuously agitated or stirred while heated/melted to allow the released volatiles/gases to escape from the melted glass and minimise the bubbles in the structure.
- Different heating rates associated with different input powers can be utilised for other purposes. For 3D printing and oxygen extraction, 1000 W input power results in better energy efficiency, faster sintering/melting times and more homogeneous products. On the other hand, lower input power with longer heating times (250 W) may concentrate Fe in SP_Fe,_ which could facilitate iron beneficiation and extraction.

Compared to the previous work under atmospheric conditions, the innovative aspect of this work is to ascertain the microwave heating behaviour of lunar soil under vacuum conditions. The findings from this work are relevant and important for addressing the ongoing development of the Microwave Heating Demonstrator (MHD) payload, which could be part of lunar ISRU demonstration missions. Furthermore, because of the volumetric heating intrinsic to the microwave heating process, the microwave heating method could be utilised for industrial-scale lunar construction and resource extraction, which is planned in the ESA’s Terrae Novae 2030+ strategy roadmap.

## Methods

### Experiment material: Lunar soil simulant JSC-1A and OPRH3N

The two materials used for this experiment are lunar soil simulant powders. Lunar mare soil simulant JSC-1A, developed by NASA’s Johnson Space Centre (JSC), is composed of particles of basaltic glass (49.3 area%), containing the minerals olivine (9.0 area%), plagioclase feldspar (37.1 area%), titaniferous magnetite (0.4 area%), pyroxene (< 0.1 area%), and ilmenite (< 0.1 area%)^9,10^. Lunar Far highland soil simulant OPRH3N, developed by Off-Planet Research, consists of 80% anorthosite and 20% basalt, 10% more anorthosite than the lunar Near highland simulant OPRH2N. While JSC-1A and OPRH3N simulants closely resemble the real lunar soil in many aspects, they cannot replicate some unique lunar features such as the nano-phase iron (np-Fe^0^) globules found on the rims of agglutinating glass and vapour-deposited glass caused by space weathering^11^. The bulk chemical compositions of JSC-1A^12^, OPRL2NT^13^, OPRH3N^14^, and NU-LHT-2M^15^ are well summarised in the cited references. OPRL2NT is a lunar mare soil simulant with higher titanium (10% anorthosite, 75.6% basalt and 14.4% ilmenite) developed by Off-Planet Research.

JSC-1A, which represents lunar mare soil, absorbs microwave energy very well due to its dielectric properties, which dictate the absorption of microwave energy. Because the lunar mare soil/simulant has a higher dielectric loss tangent and more significant dielectric loss than lunar highlands soil/simulant, it heats better with microwave heating^3^. The iron content of lunar soil/simulants significantly changes the dielectric properties of the material. Also, it indicates that JSC-1A (10–11.15 wt%) should have much better microwave absorption than that of OPRH3N (2.69 wt%). To maximise the difference in the sample materials' dielectric properties that will affect microwave heating behaviour, OPRH3N, which contains less iron oxide, has been chosen instead of NU-LHT.

### Experiment apparatus settings and procedure

The experiment apparatus settings and procedure using the bespoke 2.45 GHz microwave heating equipment is mostly the same as the previous work^3^ with a few modifications below.

- *Sample mass*: 50 g initial mass for each specimen. At least five samples were fabricated for each input power, and the sample that gave the highest yield was selected for the comparative analysis.
- *Microwave input power with total energy*: The total input energy set for this experiment was the same as the previous experiment, i.e., 900 kJ (900 s of heating time for 1000 W, 1500 s for 600 W, and 3600 s for 250 W). However, due to the lower heating performance under vacuum conditions explained earlier, the 1000 W OPRH3N sample was heated for 60 min (3600 kJ), four times longer than JSC-1A.
- *Temperature measurement*: The temperatures of the samples were measured at a 5-min interval. The cylindrical microwave chamber has two RAYTEK miniature Infrared sensors with fixed positions. The temperature range of the left pyrometer is between ambient to 1000 °C protected using gallium glass, while the right pyrometer’s range is between 500 and 1400 °C protected using fused quartz glass. Two support crucibles and one alumina plate (1 cm) were used to position the samples on the optimal hotspot; thus, the two pyrometers measured the side surfaces of the crucible. This means the recorded temperatures are considerably lower than the surface temperature of the samples.
- *Sample analysis:* The microstructures and chemical modifications of the thermally treated (sintered/molten) samples were imaged (Figs. 2, 3, 4 and 5) and analysed with a Scanning Electron Microscope (SEM) in Backscattered Electron (BSE) mode using an FEI Quanta 200 Scanning Electron Microscope equipped with an Oxford Instruments Energy Dispersive Spectroscopy (EDS) detector, and newly installed Zeiss CROSSBEAM 550 fitted with an Ultim-Extreme for resolution EDS and an Ultim-Max 170 mm^2^ EDS detector. The hardness and elastic modulus of the specimens were measured using a NANOINDENTER XP (MTS, USA). Indentations were performed using a diamond-tipped Berkovich indenter. The testing temperature was maintained within 20–22 °C to reduce thermal drift. For each surface location tested, 64 separate indentations were performed over dimensions 70 × 70 µm. Specimens were indented at a strain rate of 0.05 s^−1^ to a maximum depth of 500 nm. Elastic modulus and hardness were calculated using the Oliver-Pharr method, in which a second-order polynomial is fit to the unloading section of the load–displacement data. The Poisson’s ratio of the material was assumed to be 0.3. True density analysis of each specimen was performed by measuring the broken fragments of each sample generated via microwave heating using the Micromeritics AccuPyc II 1340 Gas Pycnometer. The volume of each sample was calculated using the Archimedes principle 20 times for each sample. The volume was used in conjunction with the specimen mass to calculate the sample density.

## Data Availability

All datasets generated and/or analysed during the current study are available from the corresponding author upon reasonable request.
